# Claudin-5 is involved in breast cancer cell motility through the N-WASP and ROCK signalling pathways

**DOI:** 10.1186/1756-9966-31-43

**Published:** 2012-05-04

**Authors:** Astrid Escudero-Esparza, Wen G Jiang, Tracey A Martin

**Affiliations:** 1 Metastasis and Angiogenesis Research Group, Institute of Cancer and Genetics, Cardiff University School of Medicine, Cardiff, UK; 2 Metastasis and Angiogenesis Research Group, Institute of Cancer and Genetics, Cardiff University School of Medicine, Heath Park, Cardiff, CF14 4XN, UK

**Keywords:** Breast cancer, Claudin-5, MDA-MB-231, N-WASP, ROCK

## Abstract

**Background:**

Recent studies have shown dysregulation in TJ structure of several cancers including breast. Claudin-5 is a protein member of the TJ structure expressed in both endothelial and epithelial cells. This study examined the level of expression and distribution of Claudin-5 in human breast cancer tissues and the effect of knockdown and forced expression of Claudin-5 in the MDA-MB-231 breast cancer cell line.

**Methods:**

Immunohistochemistry and quantitative-PCR were used to analyse patient tissue samples. The Claudin-5 gene was cloned and overexpressed or knocked down using ribozyme technology in human breast cancer cells. Changes in function were assessed using *in vitro* assays for invasion, growth, adhesion, wounding, motility, transepithelial resistance and electric cell-substrate impedance sensing. Changes in cell behaviour were achieved through the use of Hepatocyte Growth factor (HGF) which we have shown to affect TJ function and expression of TJ proteins. In addition, an *in vivo* model was used for tumour growth assays. Results data was analyzed using a Students two sample t-test and by Two-way Anova test when the data was found to be normalized and have equal variances. In all cases 95% confidence intervals were used.

**Results:**

Patients whose tumours expressed high levels of Claudin-5 had shorter survival than those with low levels (p = 0.004). Investigating *in vitro* the effect of altering levels of expression of Claudin-5 in MDA-MB-231cells revealed that the insertion of Claudin-5 gene resulted in significantly more motile cells (p < 0.005). Low levels of Claudin-5 resulted in a decrease in adhesion to matrix (p < 0.001). Furthermore, a possible link between Claudin-5 and N-WASP, and Claudin-5 and ROCK was demonstrated when interactions between these proteins were seen in the cells. Moreover, followed by treatment of N-WASP inhibitor (Wiskostatin) and ROCK inhibitor (Y-27632) cell motility was assessed in response to the inhibitors. Results showed that the knockdown of Claudin-5 in MDA-MB-231 masked their response after treatment with N-WASP inhibitor; however treatment with ROCK inhibitor did not reveal any differences in motility in this cell line.

**Conclusions:**

This study portrays a very new and interesting role for Claudin-5 in cell motility involving the N-WASP signalling cascade indicating a possible role for Claudin-5 in the metastasis of human breast cancer.

## Background

Metastasis is the presence of disease at distant sites due to the spread of cancer cells which results is overwhelming mortality in patients with cancer accounting for almost 90% of all cancer related deaths [1]. The process of cancer metastasis consists of linked sequential steps, so called metastatic cascade, including detachment, invasion, intravasation, circulation, adhesion, extravasation, and growth in distant organs. Extensive interactions between tumours cells and surrounding tissues during their dissemination complicate the analysis of signalling events during the cascade. Due to its complex nature, the understanding of the cellular and molecular factors is limited [2]. Most cancers, including breast cancer, originate from epithelial tissues and are characterized by abnormal and uncontrolled growth as well as presenting disorders in cell communication. Additionally underlying changes include changes in cell-cell and cell-substrate adhesion, a fundamental step allowing cancer cells to spread and ultimately metastasise. In order to activate the metastatic cascade, cancer cells must acquire a motile phenotype. Cell motility is orchestrated by a variety of complicated signal pathways, most of which are just starting to be unravelled. Motility occurs in response to chemokines or growth factor signals. In response to these stimuli, changes in the cytoskeleton, in the cell-cell adhesion structures and in the extracellular matrix (ECM) take place resulting in a motile cell capable of gaining access to the systematic circulation and ultimately metastasis [3].

Studies have shown that several Tight Junction (TJ) components are directly or indirectly involved in breast cancer progression and metastasis [4-8]. TJ are highly regulated areas of adhesion between cells. They are the most apical component of the lateral plasma membrane and create a regulated paracellular barrier to the movement of ions, solutes and immune cells between the cells and signalling pathways that communicate cell position, limit growth and apoptosis [9]. Claudins are members of the network of proteins that constitute the TJ structure. The main role of Claudins is in the regulation of paracellular selectively to small ions through the pores that themselves are capable of forming [10]. However, new roles for Claudins have challenged the idea that Claudins function only as sealing proteins. Claudins have now been shown to be involved in cellular growth and in epithelial-mesenchymal transition (EMT) [11]. These results suggest that Claudins play multiple roles beyond acting as a “doorman” in the paracellular barrier opening a new field of research. Most epithelial and endothelial cells express a mixture of different Claudin proteins and more than two different Claudin members are co-expressed in a single cell [12]. Claudin proteins are co-polymerised to form TJ strands as heteropolymers, and in a homophilic manner, between two molecules of the same Claudin member, or heterophilic manner between two different Claudin members [13]. The Claudin family is composed of more than 20 members in mammals of around 22 to 27 kDa. Claudins were first identified by Furuse *et al.*, using the same isolated fraction from chicken liver from which Occludin was first identify by Tsukita’s group in 1989 [14]. They showed for the first time that a group of proteins existed with similar sequence to each other and with four transmembrane domains where the N- and C- terminal domains are orientated towards the cytoplasm, but with no similarity to Occludin. At their C-termini, Claudins generally have a valine residue and all members have a PDZ domain that allows them to interact with other proteins in the TJ such as ZO-1, -2, and -3, MUPP, and PATJ. The interaction with cytoplasmic plaque proteins such as ZO-1 links Claudins to the actin cytoskeleton [15].

Claudin-5 was firstly described by Morita *et al.*[16]. It was initially identified as a deleted protein in patients who suffer from the velo-cardio-facial syndrome hereditary disease and was termed TMVCF (transmembrane protein deleted in velo-cardio-facial syndrome) and the gene was mapped to chromosome 22q11. It has been described as being expressed in the brain, lung and endothelial cells of the blood vessels concluding that Claudin-5 was an endothelial-specific component of the TJ strand [16]. However, several studies have reported Claudin-5 to be expressed in certain epithelial TJs, such as, the stomach, rat liver and pancreas [17] as well as in cell lines like HT-29/B6, an epithelial cell derived from human colon [18]. Studies focusing on blood-brain barrier (BBB) have proposed a “sealing” role for Claudin-5 [19,20]. Claudin-5 knock down mice were generated have shown a normal development and morphology of blood vessels in the brain, however, in terms of the barrier function, these endothelial cells showed an unexpected feature: a size-selective loosening of the BBB, in other words, only small molecules (<800 Da) were allowed to pass across the TJ but no larger molecules were affected. Moreover, Claudin-5 deficient mice died within 10 hours of birth [20]. Therefore, it appears that loss of Claudin-5 from the TJ complexes in the brain can compromise barrier function making it “leakier” while keeping their structural integrity.

Previous work from Martin *et al*. studied the expression of different TJ molecules in breast cancer leading to this current study examining the effect of Claudin-5 over-expression and knockdown in human breast cancer cells and the expression and distribution of Claudin-5 in human breast cancer tissues [21,22]. Following confirmation of the levels of expression, the cells were used in a number of *in vitro* and *in vivo* experimental assays in order to clarify a possible role of Claudin-5 in breast cancer progression. Additionally, Claudin-5 was examined in response to Hepathocyte Growth Factor (HGF) as we know that HGF modulates the function of TJ and the expression of several TJ molecules including Claudin-5 [21], and a possible role of Claudin-5 on control of cell motility involving the N-WASP and ROCK signalling pathways was revealed.

## Methods

### Reagents and antibodies

Mouse anti-Claudin-5 (H00007122-A01) was obtained from Abnova (Abnova GmbH, Heidelberg, Germany), rabbit anti-Claudin-5 (sc-28670) from Santa-Cruz Biotechnologies Inc. (Santa Cruz, USA), anti-actin (sc-8432) from Santa-Cruz Biotechnologies Inc. (Santa Cruz, USA), goat anti-N-WASP (sc-10122) from Santa-Cruz Biotechnologies Inc. (Santa Cruz, USA), mouse anti-ROCK 1 (sc-17794) from Santa-Cruz Biotechnologies Inc. (Santa Cruz, USA), secondary antibody anti-mouse peroxidase conjungated (A-9044) from Sigma (Sigma-Aldrich, Dorset, UK), secondary antibody anti-goat peroxidase conjungated (A-5420) from Sigma (Sigma-Aldrich, Dorset, UK) secondary antibody anti-rabbit peroxidase conjungated (A-6154) from Sigma (Sigma-Aldrich, Dorset, UK). N-WASP inhibitor Wiskostatin (681660-1 MG) from Calbiochem (Gibbstown, USA) and ROCK inhibitor Y-27632 (sc-3536) from Santa-Cruz Biotechnologies Inc. (Santa Cruz, USA) were used in the study.

### Cell lines and culture conditions

The human breast cancer cell line MDA-MB-231 was routinely maintained in Dulbecco’s Modified Eagle Medium (DMEM) (Sigma-Aldrich, Dorset, UK) supplemented with 10% fetal calf serum (FCS), penicillin and streptomycin (Sigma-Aldrich, Dorset, UK). The cells were incubated at 37°C, 5% CO_2_ and 95% humidity.

### Human breast specimens

A total of 133 breast samples were obtained from breast cancer patients (106 breast cancer tissues and 27 associated background or related normal tissue), with the consent of the patients and approved by the ethical committee. The pathologist verified normal background and cancer specimens, and it was confirmed that the background samples were free from tumour deposit. These tissues after mastectomy were immediately frozen in liquid nitrogen.

### Over-expression of Claudin-5 in MDA-MB-231 breast cancer cells

A range of normal human tissues were screened for Claudin-5. Normal placenta tissue was chosen for endogenous expression of Claudin-5. The human breast cancer cell line MDA-MB-231was chosen for introduction of the Claudin-5 gene. The gene, after amplification from placenta tissue cDNA was cloned into aPEF6/V5-His TOPO TA plasmid vector (Invitrogen Ltd., Paisley, UK) breast cancer cells or MDA-MB-231. Expression of the gene was confirmed by RT-PCR. The Claudin-5 expression construct and empty plasmid were, respectively, used to transfect MDA-MB-231 cells by electroporation. Stably transfected cells were then used for subsequent assays after being tested at both transcriptional and translational level. Those cells containing the expression plasmid and displaying enhanced Claudin-5 expression were designated MDA-MB-231^CL5exp^/MDA^CL5exp^, those containing the closed pEF6 empty plasmid and used as control cells were designated MDA-MB-231^pEF6^/MDA^pEF6^ and unaltered wild type were designated MDA-MB-231^WT^/MDA^WT^.

### Generation of Claudin-5 ribozyme transgenes

Antihuman Claudin-5 hammerhead ribozymes were designed based on the predictive secondary mRNA structure using Zuker’s RNA mFold program as previously reported [23]. Those knockdown cells displaying low levels of Claudin-5 were designated MDA-MB-231^CL5rib2^/MDA^CL5rib2^.

### RNA extraction and Reverse Transcription-Polymerase Chain Reaction (RT-PCR)

Cells were grown to confluence in a 25 cm^3^ flask before RNA was extracted using total RNA isolation (TRI) reagent and following the protocol provided (Sigma-Aldrich, Dorset, UK). RNA was converted to cDNA using iScript cDNA synthesis kit (Primer Desing Ltd., Southampton, UK). Following cDNA synthesis, samples were probed using actin primers to check the quality of the cDNA and confirm uniform levels within each sample together with those specific for the Claudin-5 transcript (full primer sequences are outline in Table 1). Conventional PCR was performed using a T-Cy Thermocycler (Breacon Technologies Ltd., The Netherlands) using REDTaq® ReadyMix™ PCR Reaction mix (Sigma-Aldrich, Dorset, UK). Cycling conditions were as followed: 94°C for 5 min, 94°C for 30 s, 55°C for 30 s, 72°C for 30 s and the final extension phase at 72°C for 7 min for 36 cycles. The PCR products were separated on a 2% agarose gel and electrophoretically separated. The gel was then stained with ethidium bromide prior to examine under ultraviolet light and photographs taken.

**Table 1:** Primer sequences used in this study

**Expression product**	**Primer name**	**Expression primer sequence (5′-3′)**	**Predicted size (bp)**
Claudin-5	CL5expR1	GACGTAGTTCTTCTTGTCGT	547
	CL5expF2	ATGGGGTCCGCAGCGTTGGAGATCCT	
CL5 ribozyme1	CL5ribF1	ACTAGTCCGCAGCGTTGGAGATTTCGTCCTCACGGACT	99
	CL5ribR1	CTGCAGACAGCACCAGGCCCAGCTGATGAGTCCGTGAGGA	
CL5 ribozyme2	CL5ribF2	CTGCAGCAGGTGGTCTGCGCCGTCACCTGATGAGTCCGTGAGGA	102
	CL5ribR2	ACTAGTGACCGCCTTCCTGGACCACAACATTTCGTCCTCACGGACT	
β-actin	BACTF	ACTGAACCTGACCGTACA	580
	BACTR	GGACCTGACTGACTACCTCA	

### Real-time quantitative Polymerase Chain Reaction (Q-PCR)

The assay was based on the Amplifluor system. It was used to detect and quantify transcript copy number of Claudin-5 in tumour and background samples. Primers were designed by Beacon Designer software, which included complementary sequence to universal Z probe (Intergen, Inc.). Each reaction contains 1 pmol reverse primer (which has the Z sequence), 10 pmol of FAM-tagged universal Z probe (Intergen, Inc.) and cDNA (equivalent to 50 ng RNA) (primer sequences are shown in Table 1). Sample cDNA was amplified and quantified over a large number of shorter cycles using an iCycler^IQ^ thermal cycler and detection software (BioRad laboratories, Hammelhempstead, UK) under the following conditions: an initial 5 minute 94°C period followed by 60 cycles of 94°C for 10 seconds, 55°C for 15 seconds and 72°C for 20 seconds. Detection of GAPDH copy number within these samples was later used to allow further standardisation and normalisation of the samples.

### SDS-PAGE, Western blotting and co-immunoprecipitation

MDA-MB-231 cells were grow to confluence, detached and lysed in HCMF buffer containing 0.5% SDS, 0.5% Triton X-100, 2 Mm CaCl_2,_ 100 μg/ml phenylmethylsulfonyl fluoride, 1 mg/ml leupeptin, 1 mg/ml aprotinin and 10 Mm sodium orthovanadate for 1 hour, sample buffer was added and the protein boiled at 100°C for 5 min before being spun at 13,000 g for 10 min to remove insolubles. Protein concentration was quantified using Bio-Rad Protein Assay kit (Bio-Rad Laboratories, Hertfordshire, UK). Equal amounts of protein from each cell sample were added onto a 10% or 15% (depending on protein size) acrylamide gel and being subjected to electrophoretic separation. The proteins were transferred onto nitrocellulose membranes which were blocked and probed with specific primary antibodies (1:500), following with peroxidase-conjugated secondary antibody (1:1000). Protein bands were visualized with Supersignal West Dura system (Perbio Science UK Ltd., Cramlington, UK) and detected using a CCD-UVIprochemin system (UVItec Ltd., Cambridge, UK).

Co-immunoprecipitation samples were prepared as follows: cell lysate of the protein of interest was probed with primary antibody (1:100 dilution) and placed on a rotating wheel for 2 hour allowing Claudin-5 antibody to bind to their targets. One hundred microlitres of conjugated A/G protein agarose beads (Santa-Cruz Biotechnologies Inc., USA) were added to each sample to make the antibody-protein complex insoluble, followed by overnight incubation on the rotation wheel. The supernatant was discarded and the pellet was washed in 200 μl of lysis buffer and resuspended in 200 μl of 2X Lamelli sample buffer concentrate (Sigma-Aldrich, Dorset, UK), then denatured for 5 minutes by boiling at 100°C. Two Claudin-5 antibodies were used to prevent cross-reactivity with N-WASP and ROCK antibodies.

### Trans-epithelial resistance (TER)

Cells were seeded into 0.4 μm transparent pore size inserts (Greiner bio-one, Stonehouse, UK) at a density of 50,000 cells in 200 μl of ordinary medium within 24 well plates, grown to confluence, the medium removed and replaced with fresh Dulbecco’s Modified Eagle’s medium containing 15 Mm Hepes, L-Glutamine ( Lonza Laboratories, Verviers, Belgium). Medium alone was added to the base of the wells (control) or with 50 ng/ml HGF [22]. Resistance across the layer of MDA-MB-231 cells was measured using an EVON volt-ohmmeter (EVON, World Precision Instruments, Aston, Herts, UK), equipped with static electrodes (WPI, FL, USA) for a period of 4 h.

### *In vitro* cell growth assay

MDA-MB-231 cells were seeded into a 96 well plate at a density of 3,000 cells/well to obtain density readings after 4 hours (day 0), 1 day, 3 days and 4 days. Within each experiment four duplicates were set up. After appropriate incubation periods, cells were fixed in 4% formaldehyde in BSS for 5-10 minutes before staining for 10 minutes with 0.5% (w/v) crystal violet in distilled water. The crystal violet was then extracted from the cells using 10% acetic acid. Absorbance was determined at a wavelength of 540 nm on a plate reading spectrophotometer.

### *In vitro* cell matrix adhesion assay

The cell-matrix attachment was carried out as previously described method [24]. Briefly, 45,000 cells were seeded onto the Matrigel basement (10 μg/well) membrane in 200 μl of normal medium and incubated at 37°C with 5% CO_2_ for 40 minutes. After the incubation period, the medium was aspirated and the membrane washed 5 times with 150 μl of BSS to remove the non-attached cells, then fixed in 4% formaldehyde (v/v) in BSS for 10minutes before being stained in 0.5% crystal violet (w/v) in distilled water. The number of adherent cells were counted from 5 random fields per well and 5 duplicate wells per sample, under a microscope.

### *In vitro* invasion cell assay

Cell culture inserts (BD Falcom^TM^ Cell Culture Inserts, BD Bioscience, Erembodegem, Belgium) were placed into a 24-well plate using forceps and coated in Matrigel. The working solution of Matrigel was prepared at a concentration of 0.5 mg/ml in PCR water, adding 100 μl to each insert and allowing to dry overnight [25]. Once dried the inserts were rehydrated in 100 μl sterile water for 1 hour. The water was then aspirated and cells were seeded in the inserts over the top of the artificial basement membrane at a density of 30.000 cells in 200 μl per well. The plates were then incubated for 3 days at 37°C with 5% CO_2._ After the incubation period, the Matrigel layer together with the non-invasive cells was cleaned from the inside of the insert with a tissue paper. The cells which had migrated through the Matrigel and porous membrane were fixed in 4% formaldehyde (v/v) in BSS for 10 minutes before being stained in 0.5% crystal violet (w/v) in distilled water. The cells were then visualized under the microscope under X40 magnification, 5 random fields counted and duplicate inserts were set up for each test sample.

### *In vitro* Cytodex-2-bead motility assay

Cells were pre-coated onto Cytodex-2 beads (GE Healthcare, Cardiff, UK) for 2 hours [26]. The medium was aspirated and the beads were washed 2X in growth medium to remove non-adherent or dead cells. After the second wash the beads were resuspended in 5 ml of normal growth medium. Cell were aliquoted into a 24-well plate, 5 duplicate wells per sample (300 μl/well), and incubated overnight. Following incubation, any cells that had migrated from the Cytodex-2 beads and adhered to the base of the wells were washed gently in BSS, fixed in 4% formaldehyde (v/v) in BSS for 10 minutes before being stained in 0.5% crystal violet (w/v) in distilled water. Five random fields per well were counted under microscope.

### Wound healing assay

Forty thousand cells were seeded in a 24 well plate, and upon reaching confluence, the medium was changed and the monolayer was scraped with a fine gauge needle to create a wound. The plate was placed on a heated plate to keep a constant temperature of 37°C. Cells were photographed after wounding and every 15 minutes during 1 hour with a CCD camera attached to a microscope at X20 magnification [27].

### ECIS

The 1600R model of the ECIS (electric cell-substrate impedence sensing) instrument (Applied Biophysics Inc, NJ, USA) was used for motility assay (wounding assay), wounding/cell modelling analysis in the study model. The ECIS instrument measures the resistence/impedance and capacitance of cells attached to a gold electrode. Cell modelling was carried out using the ECIS RbA modelling software, supplied by the manufacturer .The 8 W10 arrays (8 well format with 10 probes in each well) were used in the present study. The array surface was treated with 200 μl of 10 mM L-Cysteine solution for 20 minutes, which binds to the gold surface via its thiol group forming a monomolecular layer, followed by two washes in Dulbecco’s Modified Eagle’s medium with 15 Mm Hepes, L-Glutamine (Lonza Laboratories, Verviers, Belgium). An electrode check was run to check the impedance value of the cell-free wells containing just fresh medium and to assess the integrity of the arrays. The arrays were seeded at a density of 40,000 cells in 400 μl of Dulbecco’s Modified Eagle’s medium with 15 Mm Hepes, L-Glutamine to achieve confluent monolayers following treatment with motility-related inhibitors. After 24 hours in culture, the confluence and viability of the cell monolayer was confirmed by a light microscope, thus another electrode check was run to check the impedance value of the array to ensure correct position of the contacts [27]. The monolayer of MDA-MB-231 cells was electrically wounded with a 5 V AC at 4,000 Hz for 30 seconds. Impedance and resistance of the cell layer were immediately recorded every millisecond for a period of up to 5 hours.

#### Immunohistochemistry

Cryostat sections of frozen tissue were cut at 6 μm, placed on Super Frost Plus slides (LSL UK, Rochdale, UK), air dried and fixed in a 50:50 solution of alcohol:acetone. The sections were then air dried again and stored at -20°C until used. Immediately before commencement of immuno-staining, the sections were washed in buffer for 5 min and treated with horse serum for 20 min as a blocking agent to non-specific binding. Sections were stained using Claudin-5 antibodies (Santa-Cruz Biotechnologies Inc., Santa Cruz, USA). Negative controls were used where necessary. Primary antibodies were used at 1:100 dilution for 60 min and then washed in buffer. The secondary biotinylated antibody at 1:100 dilution (Universal secondary, Vectastain Elite ABC, Vector Laboratories Inc., Burlingham, CA, USA) was added (in horse serum/buffer solution) for 30 min, followed by numerous washings. Avidin/Biotin complex was added for 30 min, again followed with washes. Diaminobenzadine was used as a chromogen to visualize the antibody/antigen complex. Sections were counterstained in Mayer’s haematoxylin for 1 min, dehydrated, cleared and mounted in DPX.

### *In vivo* development of mammary tumour

Athymic nude mice (nu/nu) were purchased from Charles River Laboratories (Charles River Laboratories, Kent, UK) and maintained in filter top units according to Home office regulation. Each group of mice consisted of 5 mice and each mouse was injected with a mix of 2x10^6^ cancer cells in 100 μl of sterile BSS containing 0.5 mg/ml Matrigel suspension in both flanks. Two groups were included: MDA-MB-231^pEF6^ control transfected cells, and MDA-MB-231^CL5exp^ displaying enhanced Claudin-5 expression. The mice were weighted and the size of the growing tumour measured using vernier callipers under sterile conditions every week. Those mice that developed tumours exceeding 1 cm^3^ or suffered 25% weight loss during the experiment were terminated under Schedule 1 according to the UK Home Office and the UK Coordinating Committee on Cancer Research (UKCCCR) instructions. At the end of the experimental work, animals were weighed, terminated under Schedule 1 and tumours were removed if of sufficient size. Tumour volume was determined, at each point, using the following formula: tumour volume = 0.523 x width^2^ x length.

### Statistical analysis

Results data was analyzed using SigmaPlot software (version 11.0). The statistical comparisons between the test (MDA-MB-231CL5exp/MDACL5exp and MDA-MB-231CL5rib2/MDACL5rib2) and the control cell line, using as control wild type cells (MDA-MB-231WT/MDAWT) or cells containing a closed pEF6/ V5-His TOPO TA plasmid vector (MDA-MB-231pEF6/MDApEF6) were made using a Students two sample t-test and by Two-way Anova test when the data was found to be normalized and had equal variances. Comparisons between different patients groups were made using two sample t-test where appropriate. In order to assess the long term survival rates of patients with high and low levels of Claudin-5, the overall survival data was used to plot Kaplan-Meier survival curves (SPSS version 14).

## Results

### Claudin-5 expression was correlated with long-term survival

**Figure 1:**
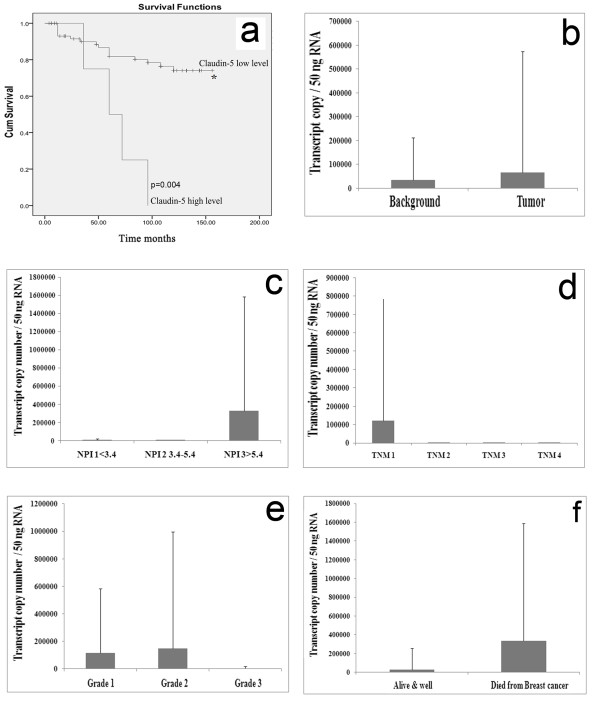
**Patients with breast cancer and high levels of Claudin-5 have reduced survival.** (**a**) Patients with high levels of Claudin-5 transcript correlates with a significantly shorter survival (p = 0.004). (**b**) Claudin-5 mRNA level was increased in human breast tumors**.** (**c**) Claudin-5 transcript levels were increased in patients with poor prognosis (NPI 3). (**d**) Higher levels of Claudin-5 transcripts were seen in at TNM1. (**e**) Claudin-5 transcript levels were decreased in grade 3 when compared to grade 1 and grade 2. (**f**) Patients who died of breast cancer had higher levels of Claudin-5 transcript when compared with patients who remained disease free.

The expression of Claudin-5 was examined in breast cancer specimens (tumour, n = 106; background, n = 27) using real-time quantitative Polymerase Chain Reaction (all values are displayed as mean Claudin-5 transcript copies/μL of cDNA from 50 ng total RNA and normalised by GAPDH). Initially long-term survival was analysed using Kaplan-Meier survival curves (Figure 1a). Patients were classified according to expression levels of CL-5, guided by the Nottingham Prognostic Index (NPI) into two groups; those with high levels and those with low levels of Claudin-5. The cut off point was set at the level at which patients were classified as moderate prognoses or NPI 2Patients with high levels of Claudin-5 transcript had a significantly shorter survival than patients with low levels of Claudin-5 (p = 0.004); mean survival 129.780 moths (118.120-141.441 months, 95% CI) versus 66 months (41.520-90.480 months, 95% CI, cut-offs as previously determined [24]). However, results revealed no significant difference between tumour and normal/background samples (p = 0.38 (Figure 1b).

### Correlation of Claudin-5 with prognosis, staging and clinical outcome

To assess levels of expression of Claudin-5 with disease progression, Claudin-5 transcript levels in the breast cancer samples were analysed against The Nottingham Prognostic Index (NPI), tumour-node-metastasis (TNM) and histological grade. NPI, which indicates the predicted prognosis of the patients, was calculated using the following equation [NPI = (0.2 X size) ± grade ± nodal status], where NPI ≤ 3.4 is regarded as a good prognosis (NPI 1), NPI 3.4-5.4 as moderate (NPI 2) and NPI ≥5.4 as poor prognosis (NPI 3). Claudin-5 levels were increased in tumors with an NPI status of NPI3. There were higher levels of Claudin-5 expression seen in patients with poorer prognosis (Figure 1c), although this did not reach significance (p = 0.34). The levels of Claudin-5 were also analysed against tumour-node-metastasis (TNM) (Figure 1d). There were higher levels of Claudin-5 expression seen in TNM1 status when compared to TNM2 (p = 0.19), TNM3 (p = 0.19) and TNM4 (p = 0.19), but significance was not reached. When comparing the levels of Claudin-5 against tumour grade (Figure 1e), little difference in expression was observed (p ≤ 0.85).

Patients who died of breast cancer had higher levels of Claudin-5 transcript when compared with patients who remained disease free although this did not reach significance (p = 0.36) (Figure 1f).

### Distribution and expression of Claudin-5 in tumour and background breast tissues

**Figure 2:**
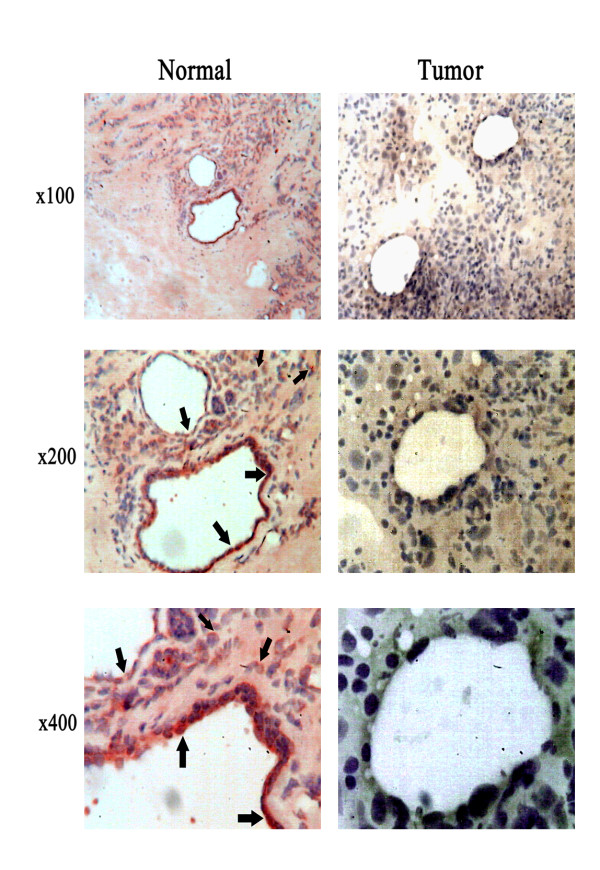
**Expression of Claudin-5 in mammary tissues Immunohistochemical staining of Claudin-5 in normal/background (left panel) tissue and tumour breast tissues (right panel) is shown in consecutively increasing magnification.** Regions of Claudin-5 expression located at the TJ area in endothelial and epithelial cells are indicated by arrows.

Claudin-5 immunohistochemical staining was observed in the human breast tissue sections compared with its staining in the normal mammary tissue (Figure 2). The staining was used to assess the location, distribution and the degree of staining of Claudin-5 in tumour and normal/background samples. In normal mammary tissues, Claudin-5 appeared as strong staining in the endothelial cells, lining vessels, whereas epithelial cells stained weakly for Claudin-5. The staining for Claudin-5 within the tumour sections was however, decreased in both endothelial and epithelial cells. Moreover, the staining distribution within cells from normal/background sections was concordant with TJ location. No such distribution was observed in cells from tumour sections. Here, the staining was weak, diffuse and not located at the TJ.

### Generation of Claudin-5 knockdown and over-expression in a human breast cancer cell line

**Figure 3:**
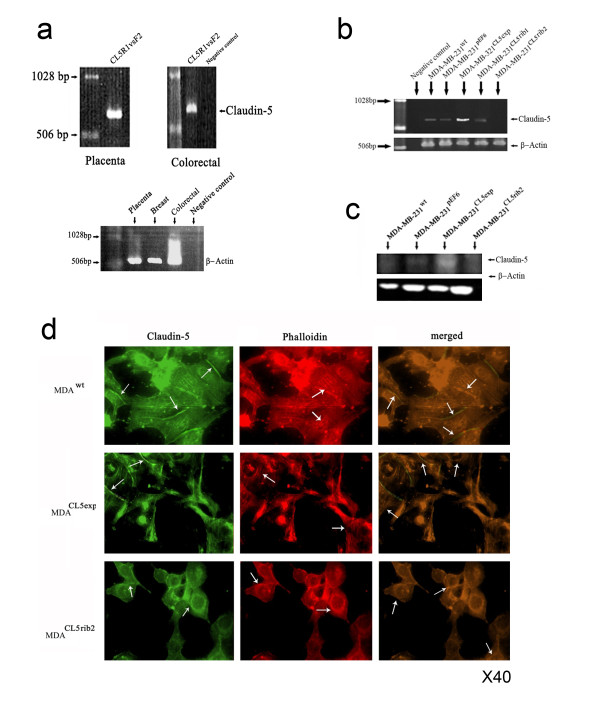
**Confirmation of over-expression and knockdown in MDA-MB-231 cells.** (**a**) Screening of different human tissues for Claudin-5 coding sequence at mRNA level using RT-PCR. β-actin is used as a loading control. The placenta tissue was selected as a template. (**b**) Verification of Claudin-5 over-expression and knockdown in MDA-MB-231cells. Claudin-5 levels were higher in MDA-MB-231 ^CL5exp^ compared to the controls, as seen at mRNA level using RT-PCR. Claudin-5 expression was reduced in MDA-MB-231 ^CL5rib2^ when ribozyme 2 was used, at mRNA level using RT-PCR. (**c**) Protein level using Western blot analysis to show expression of Claudin-5. (**d**) Immunofluorescence staining showing the distribution of Claudin-5 in Overexpressing cells (left) with Phalloidin to show actin (centre) and merged (right).

A range of human tissues were screened for Claudin-5. The Claudin-5 gene was successfully amplified from normal placenta tissue (Figure 3a). Following cloning and transfection, the human breast cancer cell line MDA-MB-231 was verified for Claudin-5 over-expression at both the mRNA using RT-PCR and protein levels using Western blot. The MDA^CL5exp^ cells demonstrated increased mRNA and protein levels of Claudin-5 compared to MDA^WT^ and empty plasmid control MDA^pEF6^ (Figure 3b).

In order to determine whether low levels of Claudin-5 has an effect on cells; ribozyme transgenes were generated to down-regulate Claudin-5 expression in this cell line. Two Claudin-5 targeting ribozyme, ribozyme 1 and ribozyme 2, were transfected into the cells together with an empty plasmid. Claudin-5 knockdown was verified at both mRNA and protein levels using RT-PCR and Western blotting (Figure 3c). However, ribozyme 1(MDA^CL5rib1^) was unsuccessful in knockdown of Claudin-5 expression; therefore only the cells expressing low levels of Claudin-5 are further referred to as MDA^CL5rib2^. The MDA^CL5rib2^ cells demonstrated reduced mRNA and protein levels of Claudin-5 compared to the controls, MDA^WT^ and MDA^pEF6^. Immunostaining revealed some increase in Claudin-5 at the cell periphery (Figure 3d).

### Claudin-5 did not alter cell growth in transfected human breast cancer cells

**Figure 4:**
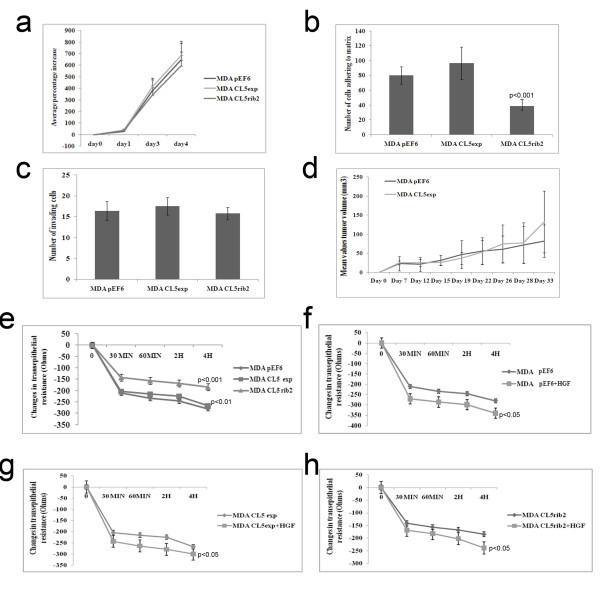
***In vitro*effect of Claudin-5 expression on and *in vivo *tumor development of MDA-MB-231 cells.** (**a**) The cell growth of MDA^Cl5exp^ and MDA^CL5rib2^ did not show any significant difference when compared to MDA^pEF6^ (mean ± SD, n = 3). (**b**) The adhesive capacity of MDA^CL5rib2^ was significantly decreased in comparison with the control MDA^pEF6^ (p ≤ 0.001) (mean ± SD, n = 3). **(c)** The invasive capacity of MDA^Cl5exp^ and MDA^CL5rib2^ did not show any significant difference when compared to MDA^pef6^ (mean ± SD, n = 3). (**d**) There were no significant differences in tumor growth over 33 day period (p = 0.29). (**e**) A significant increase was seen in TER of MDA^CL5rib2^ over a period of 4 hours when compared to the control (p ≤ 0.001) (mean±SD, n = 3). (**f**) Effect of Claudin-5 on transepithelial resistance on MDA-MB-231 cells after treatment with HGF showed significant decreases in TER in control cells MDA^pEF6^ over a period of 4 hours when compared to the untreated cells (using ANOVA p ≤ 0.05 versus respective untreated cells) (mean±SD, n = 3). (**g**) Significant decreases in TER were also seen in the transfected cells MDA ^CL5exp^ after treatment with HGF (using ANOVA p ≤ 0.05 versus respective untreated cells) (mean±SD, n = 3) and in MDA ^CL5rib2^ (**h**) (using ANOVA p ≤ 0.05versus respective untreated cells) (mean±SD, n = 3).

The MDA-MB-231 sublines MDA^Cl5exp^ and MDA^CL5rib2^ alongside MDA^pEF6^ were examined following 1, 3 and 4 day incubation periods using an *in vitro* cell growth assay. No significant difference in the *in vitro* growth rate of the MDA^pEF6^ cells compared to MDA^Cl5exp^ or MDA^CL5rib2^ were found following the three different incubation periods (Figure 4a).

### Low levels of Claudin-5 reduces the cell adhesion to an artificial Matrigel basement membrane

The ability of MDA^Cl5exp^ and MDA^CL5rib2^ cells to adhere to matrix was assessed in an *in vitro* Matrigel adhesion assay (Figure 4b). There was a significant difference between the adherence of MDA^CL5rib2^ and MDA^pEF6^ with MDA^CL5rib2^ cells being less adherent to matrix. In the case of MDA^Cl5exp^, the opposite effect was seen, however differences did not reach statistical significance when compared to the control.

### Claudin-5 did not alter the invasive phenotype of transfected human breast cancer cells

The invasive potential of the transfected cells MDA^Cl5exp^ and MDA^CL5rib2^ was examined using an in vitro Matrigel invasion assay (Figure 4c). Both cell lines were found to have no significant differences when compared to the control MDA^pEF6^ and invaded as individual cells, with no apparent difference in invasion patterns.

### Claudin-5 did not alter the *in vivo* tumour growth of human breast cancer cells

The growth and capability of developing tumours of MDA^Cl5exp^ in an *in vivo* model was examined and compared to the control MDA^pEF6^ cells after subcutaneous injection into the athymic nude mouse model. Over the period of 33 days, no significant difference was observed between the two groups, the control (injected with MDA^pEF6^) and those injected with MDA^Cl5exp^ (Figure 4d).

### Low levels of Claudin-5 confers increased trans-epithelial resistance (TER) in human breast cancer cells

Transepithelial resistance was measured to assess the effect of over-expressing or knocking-down Claudin-5 on TJ functionality in MDA-MB-231 breast cancer cells (Figure 4e). If the cells were to produce a higher resistance, this is interpreted as them having increased Tight Junction function; conversely, reduced resistance implies a loss of cell-cell contact and a reduced Tight Junction function. MDA^Cl5exp^ showed increased TER over a period of 4 hours in comparison with the control MDA^pEF6^. Changes in TER were more evident in MDA^CL5rib2^ when compared to the control. Treatment of cells with HGF (50 ng/ml) resulted in a significant reduction of the transepithelial resistance in transfected and in control cells when compare to untreated cells over a period of 4 hours (Figure 4f, g, h).

### Low levels of Claudin-5 retarded the motility and migration of human breast cancer cells

**Figure 5:**
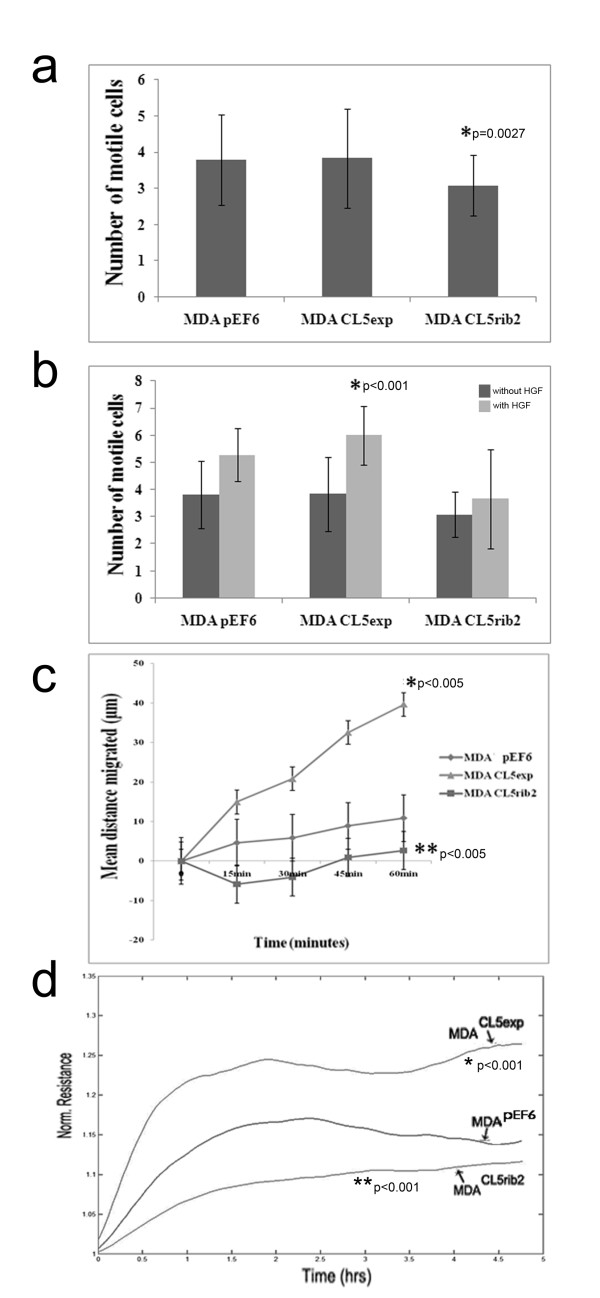
**Effect of Claudin-5 on cell motility of MDA-MB-231 cells.** (**a**) Cytodex-2 bead motility assay was used. The motility of MDA ^CL5rib2^ was significantly reduced in comparison to the control MDA ^pEF6^ (using one-tailed test, p = 0.027) (mean±SD, n = 3). (**b**) Effect on cell motility after treatment with HGF using a Cytodex-2 bead motility assay. Transfected and control cells showed an increase in motility, however only MDA ^Cl5exp^ results were significant (p ≤ 0.001 versus respective untreated cells) (mean±SD, n = 3). (**c**) Effect of Claudin-5 on cell migration was assessed by a migration/wound healing assay. MDA^CL5exp^cells showed an increase in migration when compared to the control at 60 minutes after wounding (*p ≤ 0.005) (mean ± SD, n = 3). The migration of MDA^Cl5rib2^ was reduced in comparison to the control at 60 minutes (**p ≤ 0.005) (mean ± SD, n = 3). (**d**) Significant differences using ECIS were revealed after wounding. MDA^CL5exp^ showed significant increased migration (p ≤ 0.001) whereas MDA^Cl5rib2^ showed a decreased migration rate (p ≤ 0.001) (n = 3).

Transfected and control cells, either untreated or treated with HGF, were evaluated for their motility using a Cytodex-2 bead motility assay to explore the possibility of Claudin-5 involvement in motility. The aim of this assay is to evaluate the number of motile cells that are able to detach and become motile from beads onto the culture vessel floor. MDA^Cl5exp^ cells did not show significant differences when compared to the control. In contrast, MDA^CL5rib2^ cells demonstrated a significant reduction in cell motility compared to the control (Figure 5a). The cells were additionally evaluated after treatment with HGF. This motogen increased cell motility in MDA^Cl5exp^ and control cells when compared to untreated. In the case of MDA^CL5rib2^, changes in motility were not found to be significant (Figure 5b).

The effect of Claudin-5 on cell migration was assessed using an *in vitro* cellular migration/wound healing assay. MDA^Cl5exp^ showed a significant increase in cellular migration compared to the control 60 minutes after. A significant decreased cell migration was seen in MDA^CL5rib2^ after 60 minutes when compared to control (Figure 5c). In this assay, we are investigating the direct movement of cells as they migrate from a cell layer into open space. The cytodex-2 bead assay in comparison, measures the motility of single cells. It is not surprising that the over-expression or knock-down of Claudin-5 appears to be more significant in the wounding assay; it appears that Claudin-5 might be involved in the signalling pathway for changes in contact inhibition and changes in the cytoskeleton, rather than in simple motility (as assessed using the bead assay).

Using ECIS (Electrical Cell Impedance Sensing) and in recovering from electrical wounding (5 V AC for 30 seconds), it was shown that the MDA^Cl5exp^ cells were significantly more motile compared to the control cells as the resistance in the electrode increased as the cells begin to spread over the electrode, whereas the opposite trend was seen in MDA^CL5rib2^, where a significant reduction in migration was seen (Figure 5d).

### Claudin-5 and control of cell motility involving the N-WASP and ROCK signalling pathways

**Figure 6:**
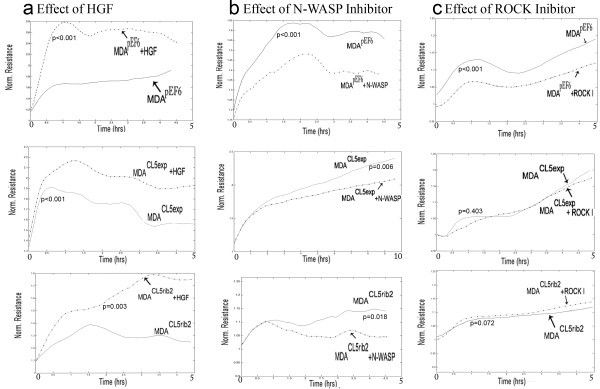
**Effect of Claudin-5 on MDA-MB-231 cell migration following treatment with HGF, N-WASP inhibitor or ROCK inhibitor using ECIS.** (**a**) Migration was significantly increased in MDA^pEF6^ ± HGF, MDA^Cl5exp^ ± HGF and MDA^CL5rib2^ ± HGF when compared to untreated cells (p ≤ 0.001, p ≤ 0.001 and p = 0.003 versus respective untreated controls) (n = 3). (**b**) Migration was significantly decreased in MDA^pEF6^ ± N-WASP inhibitor, MDA^Cl5exp^ ± N-WASP inhibitor and MDA^CL5rib2^ ± N-WASP inhibitor when compared to untreated cells (p ≤ 0.001, p = 0.006 and p = 0.018 respectively) (n = 3). (**c**) Migration was significantly decreased in MDA^pEF6^ ± ROCK inhibitor (p ≤ 0.001). MDA^Cl5exp^ ± ROCK inhibitor and MDA^CL5rib2^ ± ROCK inhibitor did not show significant differences when compared to untreated cells (p = 0.403 and p = 0.072 respectively) (n = 3).

To address the possibility that Claudin-5 might play a role in regulating cell motility, different motility-regulators were studied in order to search for any possible links between Claudin-5 and a range of motility-related proteins. Cell motility was analysed using ECIS after being treated with different motility inhibitors and the motogen HGF. Following electrical wounding (5 V AC for 30 seconds) and treatment with HGF (50 ng/ml), MDA^pEF6^ ± HGF, MDA^Cl5exp^ ± HGF and MDA^CL5rib2^ ± HGF showed an increase in motility when compared to untreated cells. It was significantly enhanced after 5 hours of treatment (Figure 6a). Following experiments then examined the effect of motility inhibitors alone. When cells were treated with the N-WASP inhibitor (50 μM), the migration rate of MDA^pEF6^ ± N-WASP, MDA^Cl5exp^ ± N-WASP and MDA^CL5rib2^ ± N-WASP was markedly reduced after 5 hours of treatment when compared to untreated cells (Figure 6b). The ROCK inhibitor (50nM) was capable of altering the motility of MDA^pEF6^ ± ROCK when compared to the untreated cells. However, no significant differences were found in the transfected cells, MDA^Cl5exp^ ± ROCK and MDA^Cl5rib2^ ± ROCK, when compared to the untreated cells (Figure 6c). All these results were based on 3 repeat experiments that were combined and analysed using ANOVA.

**Figure 7:**
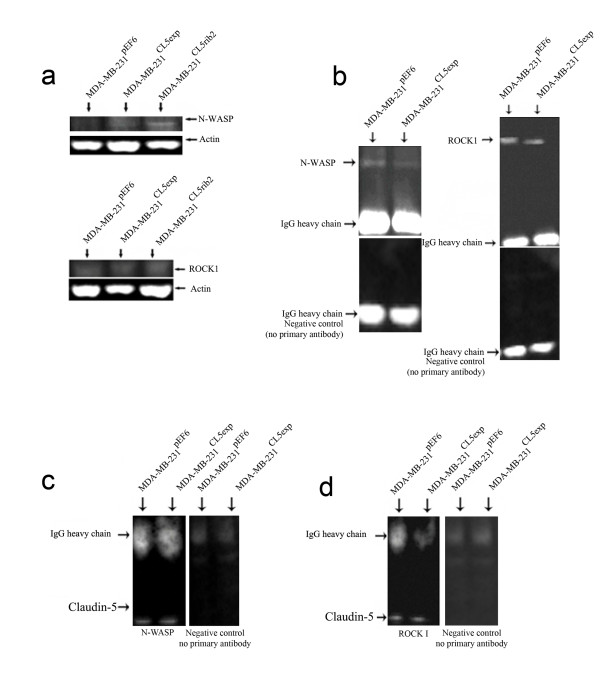
**Western blot demonstrating levels of expression of N-WASP and ROCK 1 and protein-protein interactions.** (**a**) Expression of N-WASP and ROCK 1 in transfected and control cells. (**b**) Co-immunoprecipitation of Claudin-5 with N-WASP and ROCK 1. (**c**) Co-immunoprecipitation of N-WASP with Claudin-5. (**d**) Co-immunoprecipitation of ROCK 1 with Claudin-5.

In order to investigate any possible effect of Claudin-5 on protein level of N-WASP and ROCK 1, Western blot analysis was used to assess whether any direct effect was exerted at the protein level in the control and transfected cells. MDA-MB-231^Cl5exp^ and MDA-MB-231^CL5rib2^ Western blotting demonstrated very low levels of the N-WASP at protein level which was undetectable in MDA-MB-231^pEF6^ (Figure 7a). Protein levels of ROCK 1 showed a similar low level in all cells (Figure 7a). Thus, modulation of Claudin-5 appeared to cause an increase in N-WASP expression at the protein level.

Immunoprecipitation of Claudin-5 followed by immunoblotting with N-WASP and ROCK 1 was used in order to investigate a possible interaction between Claudin-5 and N-WASP as well as with ROCK 1. Results showed a protein-protein interaction between Claudin-5 and these motility-related proteins in MDA-MB-231^pEF6^ and MDA-MB-231^Cl5exp^ (Figure 7b, negative controls shown below). In keeping with this, immunoprecipitation with either N-WASP (Figure 7c) or ROCK1 (Figure 7d) followed by immunoblotting with Claudin-5 produced consistent results.

## Discussion

In this present study, we used cells transfected with Claudin-5 expression sequence and ribozyme transgenes to assess the impact of reducing the expression of our protein of interest as well as enhancing it in order to evaluate changes in the aggressive nature of MDA-MB-231 breast cancer cells. We also demonstrated for the first time that there is a link between Claudin-5 and cell motility. The disruption of the Tight Junction (TJ) structure is a common feature of many human cancer cells. Downregulation of different TJ proteins has been linked with staging and metastatic potential in various cancers including breast [28]. Indeed, in human breast cancer, tumour tissues show truncated and/or variant signals for occludin. Knockdown of occludin resulted in increased invasion, reduced adhesion and significantly reduced TJ functions, whilst Q-RT-PCR showed occludin to be significantly decreased in patients with metastatic disease [29]. This loss of or aberrant expression has clear repercussions as to the importance of occludin in maintaining TJ integrity in breast tissues and could play a part in breast cancer development. In addition, *in vivo* and *in vitro* data has revealed that over-expression of TJ proteins in cancer cells, such as Claudin-4, leads to a decrease in invasiveness and metastases in animal models [29]. Similar conclusions were found when cells breast cancer cells overexpressing Claudin-16, showed a decrease in invasiveness and motility [26]. Since claudin-18 is overexpressed in precursor lesion PanIN and pancreatic duct carcinoma, it serves as a diagnostic marker and a target of immunotherapy [30]. The upregulation of claudin-18 by TPA in human pancreatic cancer cell lines can be prevented by inhibitors of PKCδ, PKCϵ, and PKCα, whereas the upregulation of claudin-18 by TPA in hTERT-HPDE cells is prevented by inhibitors of PKCδ, PKCθ, and PKCα. This suggests that in human pancreatic cancer cells claudin-18 is primarily regulated at the transcriptional level via specific PKC signaling pathways and modified by DNA methylation [30]. These studies have provided promising evidence that TJ proteins might serve as useful molecular targets in the prognosis of cancer. In prostate, claudin-4 was down-regulated and claudins-2, -3, and -5 were overexpressed in prostate adenocarcinomas compared with benign prostatic hyperplasia samples. Expression of claudins-1 and-7 was similar in tumours and benign prostatic hyperplasia samples. Claudin-11 was absent from all prostate samples. Overexpression of claudin 3 was associated with perineural invasion and tended to occur in advanced stages of the disease. Increased expression of Claudin-5 was marginally associated with perineural invasion. Such results suggest that alterations in claudin expression occur in prostate cancer cells, although there was no association with clinicopathological parameters [31].

Initially, the role of Claudin-5 was investigated when transepithelial electric resistance (TER) was measured. Transepithelial electric resistance (TER) is the easiest and most sensitive measure of barrier strength. MDA^CL5rib2^ showed the highest resistance, whereas the resistance of MDA^Cl5exp^ and the control were lower and followed the same trend, although MDA^Cl5exp^ was significantly higher than control cells. These preliminary results revealed that Claudin-5 was not playing a real role in keeping the cell barrier tight. In fact, the compensation of the lack of Claudin-5 could be balanced with one of the other 23 members of the Claudin family which might alter the barrier strength, therefore explaining why the knockdown cells displayed higher transepithelial resistance. The same explanation could be applied to forced-expression and the very similar trends that it shared with the control cells.

The involvement of Claudin-5 in cell growth was tested, although there appeared not to be an involvement of Claudin-5 in cell growth. Cell adhesion to extracellular matrix is fundamental in the organization of the epithelium as a continuous layer but also in the regulation of many cellular processes such as motility [32]. MDA^CL5rib2^ demonstrated a decrease in adhesion whereas MDA^Cl5exp^ appeared to increase adhesion when compared to the control cells, although these results did not reach significance. Integrins enable cancer cells to identify their surrounding extracellular matrix (ECM), and they participate in the maintenance of positional stability in normal epithelia; in breast cancer however, it has been suggested that there may be a link between integrins and metastasis [33]. The question therefore arises as to whether the absence of Claudin-5 in a cell alters levels of integrins and other adhesion-related proteins, thus changing the adhesion of the cancer cell when compared to the control. The invasiveness of the cells through the ECM did not show any relevant differences between cells over-expressing or knocking-down levels of Claudin-5. This result agrees with the data obtained in the *in vivo* experiments, where the MDA^Cl5exp^ cells were analysed for their ability to grow and develop in nude mice. Over a period of one month, no differences were found between the two groups of animals, the control (injected with MDA^pef6^) and those injected with MDA^Cl5exp^. Taking these results together, we began to speculate whether Claudin-5 might be involved in cell motility. We performed a further set of experiments to assess the level of involvement of Claudin-5 in breast cancer motility. As breast cancer cells acquire a motile phenotype, this is translated into changes in highly dynamic structures like actin filaments and cytoplasmic microtubular complex [34]. We decided to investigate the effects on motility of over-expression or knockdown of Claudin-5. To achieve this, an *in vitro* motility assay and a traditional wound healing assay was carried out, both revealing that MDA^CL5rib2^ showed a reduction in motility. Moreover, ECIS was used in order to measure in real time how fast cells migrate after wounding. Similar results were obtained; MDA^CL5rib2^ was indeed slower when compared to the control. However, MDA^Cl5exp^ cells were the fastest in each of the assays mentioned above. Until now, we have shown that knockdown of Claudin-5 expression in a breast cancer cell line resulted in a less adhesive and less motile cell phenotype when compared to controls. The opposite was seen when Claudin-5 expression was forced, resulting in a more adhesive and more motile phenotype but with no differences in invasiveness *in vivo* and *in vitro*. We might tentatively conclude from this that Claudin-5 might be a motility regulator, or at least have a role in the motility of these human breast cancer cells.

Previously, we have carried out a significant body of work on the role and effect of HGF in epithelial cancer cells. HGF is a powerful motogen able to promote proliferation, invasion, and migration of epithelial cells by binding to its tyrosine kinase receptor *c*-met [35] as well as modulating expression and function of TJ molecules in human breast cancer cell lines and decreasing trans-epithelial resistance [21]. Cells displaying enhanced or suppressed expression of Claudin-5 respond in keeping with the well established effect after treatment with HGF, showing reduced epithelial resistance and increased motility. ECIS experiments corroborated these results. It is interesting that claudin-7 expressing human lung cancer cells have been shown to have a reduced response to HGF, are less motile, and form fewer foot processes than untreated cells. In addition, cells transfected with claudin-7 dramatically decreased their invasive ability after HGF treatment. It has been shown that this is mediated through the MAPK signalling pathway since the phosphorylation level of ERK1/2 was significantly lower in claudin-7 transfected cells than in control cells [36].

To address the possibility that Claudin-5 might play a role in regulating cell motility, different motility-regulators were studied in order to search for any possible links between Claudin-5 and a range of motility-related proteins. Cell motility was analysed using ECIS after being treated with different motility inhibitors. In particular the N-WASP inhibitor (Wiskostatin) and the ROCK inhibitor (Y-27632) responded in an unexpected way in our transfected cells. Neuronal Wiskott - Aldrich syndrome protein, N-WASP, is ubiquitously expressed in mammalian tissues and it is responsible for connecting several signalling pathways to the initiation of actin assembly via the Arp2/3 complex. N-WASP has been reported to exist in a self-folded auto-inhibited conformation. When activated, conformational changes occur facilitating the interaction with the Arp2/3 complex and subsequent nucleation [37]. The Rho-associated serine-threonine protein kinase, ROCK, is ubiquitously expressed in mammalian tissues and it is directly linked, after activation, with numerous processes related to actin-myosin, such as actin cytoskeletal reorganisation and the formation of focal adhesions. It also has an important role in cell migration by promoting the contraction of the cell body and is required for tail retraction in cancer cells [38]. The transfected and control cells were treated with the N-WASP inhibitor, responsible for stabilising the auto-inhibited conformation of the N-WASP protein [39], and their rate of speed was measured using ECIS after wounding. Results showed an inhibition in their motility, however, this inhibition was marginally reduced in knockdown cells. The effect of the ROCK inhibitor (Y-27632) was also studied in our cells. The inhibitor specificity is, however, questioned as *in vitro* studies revealed that it not only exerts an inhibitory effect on ROCK proteins but also on other kinases [40]. Nevertheless, the control cells responded to its inhibition showing a lower rate of migration; conversely both transfected cells did not respond to its inhibitory effects. Thus far we have shown that the absence of Claudin-5 clearly caused an alteration in cell motility as the ROCK inhibitors were no longer inhibiting cell motility in MDA^CL5rib2^. Additionally, in the case of MDA^CL5rib2^ cells treated with N-WASP inhibitor, we observed some inhibition, but at a considerably reduced manner compared to N-WASP inhibitor in control and MDA^Cl5exp^ cells.

The next question to be addressed following the ECIS results, was to investigate any possible protein-protein interaction between Claudin-5 and N-WASP or Claudin-5 and ROCK 1 as well as whether any direct effect was occurring at the protein level of these molecules in the control and transfected cells. Co-immunoprecipitation with Claudin-5, followed by immunoblotting with either N-WASP or ROCK 1 demonstrated an interaction between Claudin-5 and N-WASP as well as with ROCK 1. To confirm these interactions, a co-immunoprecipitation with either N-WASP or ROCK 1 followed by immunoblotting with Claudin-5 was carried out confirming the interactions between these protein pairs. Previously, studies have already linked TJ with N-WASP. The intestinal epithelial cells, T84, when treated with N-WASP inhibitor showed an inhibition in the formation of TJ [41]. A more recent study using Sertoli cells linked the inhibition of N-WASP, and therefore the inhibition of Arp2/3, in the nucleation process with barrier disruption in the blood-testis barrier causing a failure of spermatic transit [40]. N-WASP protein in MDA-MB-231 human breast cancer cells has been reported to be expressed at a very low level [25]. The results obtained in the current study agree. The levels of ROCK 1 did not show any real differences among transfected and control cells, this possibly could be due to the high level of this protein found in MDA-MB-231 wild type cells as already reported [38].

This work suggests that Claudin-5 might be involved in cancer cell motility; in particular, it appears to be involved in the signalling pathway of N-WASP and ROCK. However, understanding cell motility requires detailed knowledge not only of the signalling networks, but also about their dynamics. This possible new role of Claudin-5 in breast cancer cell motility opens the door to future studies in which Claudin-5 and therefore TJ might switch from static structures to very dynamic ones, and offers an exciting glimpse into how modulation of transmembrane TJ proteins could be targeted in cancer metastasis.

Previous studies have revealed the differential expression of Claudins in human cancers [32]. Although high levels of Claudin-5 have been reported in ovarian [6], prostate [42] and lung cancers [5] and low levels in hepatocellular carcinoma [43], this is the first study to our knowledge to report levels of Claudin-5 in patients with breast cancer. We have shown for the first time that Claudin-5 is aberrantly expressed in human breast cancer and has a link to the clinical outcome of the patient. From this data we have observed that Claudin-5 expression is increased in breast tumour tissue compared to normal/background endothelial cells, however this result did not correlate with IHC staining, where levels of Claudin-5 protein appear to be higher in normal/background tissues when compared to tumour sections. This discrepancy may be due to the non-discriminatory nature of Q-PCR, as we have not been able to specifically compare the levels of Claudin-5 in endothelial cells from normal mammary tissues and breast cancer tissues. In early studies Claudin-5 was described as a protein highly expressed in endothelial cells of the blood vessels [16] this might also help us to explain the disparity founded between the IHC and Q-PCR results. Moreover, IHC is a semi-quantitative method. For the clinical point of view, one of the most interesting observations from this study is the relationship between high levels of Claudin-5 and clinical outcome. Patients who died from breast cancer had higher levels of Claudin-5 compared with patients who remained disease-free. Furthermore, patients whose tumours expressed high levels of Claudin-5 had significantly shorter survival than those with low levels of expression of Claudin-5.

There is now a body of work highlighting the potential roles of claudins in human breast cancer, where low levels of some claudins (-3, -4 and -5), termed claudin-low represent a subtype of breast cancer in patients with poor prognosis and features of mesenchymal and mammary stem cells [44]. It appears that Claudin-5 has a different role in breast cancer, functioning as a potential motility regulator. Although this does not prevent other claudins having a role in Tight Junction function itself, it appears that Claudin-5 has a more unique function. Future work would hope to unravel it’s function as distinct from other claudins’. Collectively, these findings suggest that Claudin-5 is a potential prognostic factor in patients with breast cancer, as high levels of expression are clearly associated with indicators of poor prognosis as well as with high incidence of breast cancer-related death and shorter survival of patients. This report indicates that Claudin-5 has a potential as a prognostic indicator in human breast cancer .

## Conclusions

From the data presented here, we can reveal a link between Claudin-5 and cell motility in breast cancer cells. Furthermore, Claudin-5 has potential as a prognostic tool in human breast cancer, in particular with relevance to patient survival and outcome. Many questions still need to be answered and whilst high motility phenotypes might not lead to malignant progression per se, the control of motility by Claudin-5 could be a contributing factor to metastatic disease in human breast cancer.

## Abbreviations

N-WASP, Neuronal wiskott aldrich syndrome protein; ROCK, Rho kinase; TJ, Tight Junction; ECM, Extracellular matrix; EMT, Epithelial-mesenchymal transition; TMVCF, Transmembrane protein deleted in velo-cardio-facial syndrome; BBB, Blood brain barrier; HGF, Hepatocyte growth factor; Q-PCR, Quantitative polymerase chain reaction; HCMF, Hepes buffer, calcium and magnesium free; ECIS, Electric cell-substrate impedance sensing; RT-PCR, Reverse transcriptase-polymerase chain reaction; NPI, Nottingham prognostic indicator.

## Competing interests

The authors declare that they have no competing interests.

## Authors’ contributions

AEE carried out the molecular and cell biology work and drafted the manuscript. WGJ conceived of the initial plan, designed primers and carried out Q-PCR and sourced the patient samples. TAM completed the manuscript, planned the experiments and provided additional laboratory help, carried out Q-PCR and contributed to the overall design of the work. All authors read and approved the final manuscript.
